# Is there any advantage of using stand-alone cages? A numerical approach

**DOI:** 10.1186/s12938-019-0684-8

**Published:** 2019-05-22

**Authors:** Andrea Calvo-Echenique, José Cegoñino, Amaya Perez del Palomar

**Affiliations:** Group of Biomaterials, Aragón Institute of Engineering Research (I3A), Mechanical Engineering Department, University of Zaragoza, C/Maria de Luna s/n. Betancourt Building, 50018 Saragossa, Spain

**Keywords:** Finite element method, Intervertebral disc, Lumbar spine, Stand-alone cages, Posterior screw fixation, Posterior lumbar interbody fusion

## Abstract

**Background:**

Segment fusion using interbody cages supplemented with pedicle screw fixation is the most common surgery for the treatment of low back pain. However, there is still much controversy regarding the use of cages in a stand-alone fashion. The goal of this work is to numerically compare the influence that each surgery has on lumbar biomechanics.

**Methods:**

A non-linear FE model of the whole lumbar spine was developed to compare between two types of cages (OLYS and NEOLIF) with and without supplementary fixation. The motion of the whole spine was analysed and the biomechanical environment of the adjacent segments to the operated one was studied. Moreover, the risk of subsidence of the cages was qualitatively evaluated.

**Results:**

A great ROM reduction occurred when supplementary fixation was used. This stiffening increased the stresses at the adjacent levels. It might be hypothesised that the overloading of these segments could be related with the clinically observed adjacent disc degeneration. Meanwhile, the stand-alone cages allowed for a wider movement, and therefore, the influence of the surgery on adjacent discs was much lower. Regarding the risk of subsidence, the contact pressure magnitude was similar for both intervertebral cage designs and near the value of the maximum tolerable pressure of the endplates.

**Conclusions:**

A minimally invasive posterior insertion of an intervertebral cage (OLYS or NEOLIF) was compared using a stand-alone design or adding supplementary fixation. The outcomes of these two techniques were compared, and although stand-alone cage may diminish the risk of disease progression to the adjacent discs, the spinal movement in this case could compromise the vertebral fusion and might present a higher risk of cage subsidence.
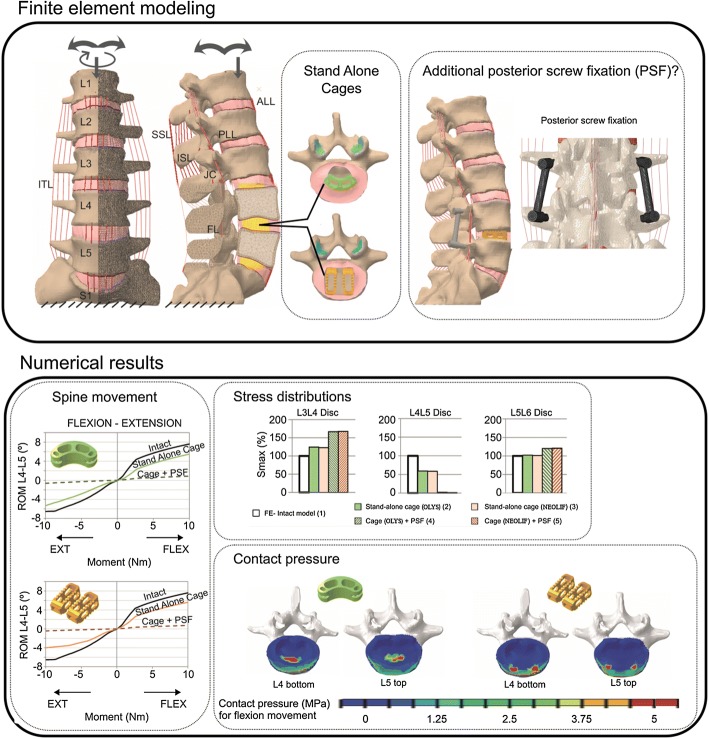

**Electronic supplementary material:**

The online version of this article (10.1186/s12938-019-0684-8) contains supplementary material, which is available to authorized users.

## Introduction

Segment fusion with intradiscal cage and pedicle screw fixation (PSF) is the “gold standard” treatment for lumbar hernia and degenerative intervertebral disc (IVD) diseases. However, stand-alone interbody cages have shown to be a feasible surgical technique for the treatment of discogenic back pain [1, 2]. A simple discectomy without cage insertion reduces the disc height creating slack in all longitudinal ligaments [3]. Nevertheless, if the IVD space is distracted by a cage insertion, the ligaments are pre-strained contributing to spine stabilisation [4]. Furthermore, using a minimally invasive approach, important stabilising structures such as posterior musculature, anterior and posterior longitudinal ligaments, and facet joints are preserved helping to control segment kinematics [5–7]. In spite of that, some physicians advocate for the use of supplementary PSF to assure long-term stabilisation and segment fusion [8, 9]. Few clinical prospective randomised studies have been comparing stand-alone construct versus fusion with supplemental PSF [10, 11]. These studies did not show significant differences in clinical outcomes whilst several advantages were reported for the use of stand-alone cages in degenerated lumbar segments without previous instability: the surgical technique is less demanding, it takes less time, the implant cost is lower and pedicle screw-related complications are avoided. Besides, different cohorts of patients undergoing a stand-alone cage implantation have been followed up showing good clinical outcomes, a high rate of fusion and a low incidence of both cage subsidence and cage migration [1, 2, 12–15]. Finally, comparisons of cages with and without different supplemental fixations have been performed in vitro in human lumbar spines [16–18].

Several computational works have been developed using finite element (FE) models to simulate lumbar biomechanics after cage insertion in single functional spinal unit (FSU) [19–21] or complete lumbar spine [22–25]. All of them studied the spinal movement showing that PSF provided a higher segment stiffness than stand-alone cages [26, 27], but segment stability was also reported for the last ones. Since the goal of lumbar surgery is not only to stabilise the segment but also to restore the IVD space and maintain the lumbar lordosis, the major concerns regarding surgery complications are segmental instability [28], cage subsidence [4] and cage migration [29]. Furthermore, lumbar fusion has been associated with the risk of adjacent segment disease because it alters the biomechanical environment of the whole spine [30]. However, as far as the authors’ knowledge, all these key factors have not been studied together in a complete lumbar spine model using complex constitutive models for the biological tissues involved.

On the other hand, ligaments play an important role in segment behaviour, particularly in bending. Ligament pre-strain is thought to be responsible for spinal stability in the absence of active muscle contraction [31]. However, ligament pretension is often overlooked or not reported in lumbar spine FE models because of the lack of experimental values. Recently, some FE studies have introduced the experimentally characterised pre-strain of some spinal ligaments in healthy lumbar spines [32, 33] showing their influence on the overall spine biomechanics. Despite the importance that the ligament pre-strain may have on lumbar surgery success, few computational works have considered this condition after cage insertion [19].

In this work, an FE model of the whole human lumbar healthy spine was developed, and then this model was modified to insert an intervertebral cage in L4–L5 segment in a stand-alone fashion or in combination with PSF. In addition, two different cage designs were compared. In addition, the role that ligament pre-stresses play in spinal behaviour was discussed in a functional spinal unit (FSU). Thus, the goal of this study was to analyse the influence that each surgery has over the biomechanics of the affected and adjacent segments and discuss the outcomes of each of them.

## Materials and methods

In this paper, the insertion of two different types of intervertebral cages at L4–L5 level with and without posterior screw fixation (PSF) is analysed. For both the scenarios (with or without PSF), a minimally invasive surgery is simulated. This means that only a discectomy is performed and an intervertebral cage is inserted, not removing any additional tissue [34].

**Fig. 1:**
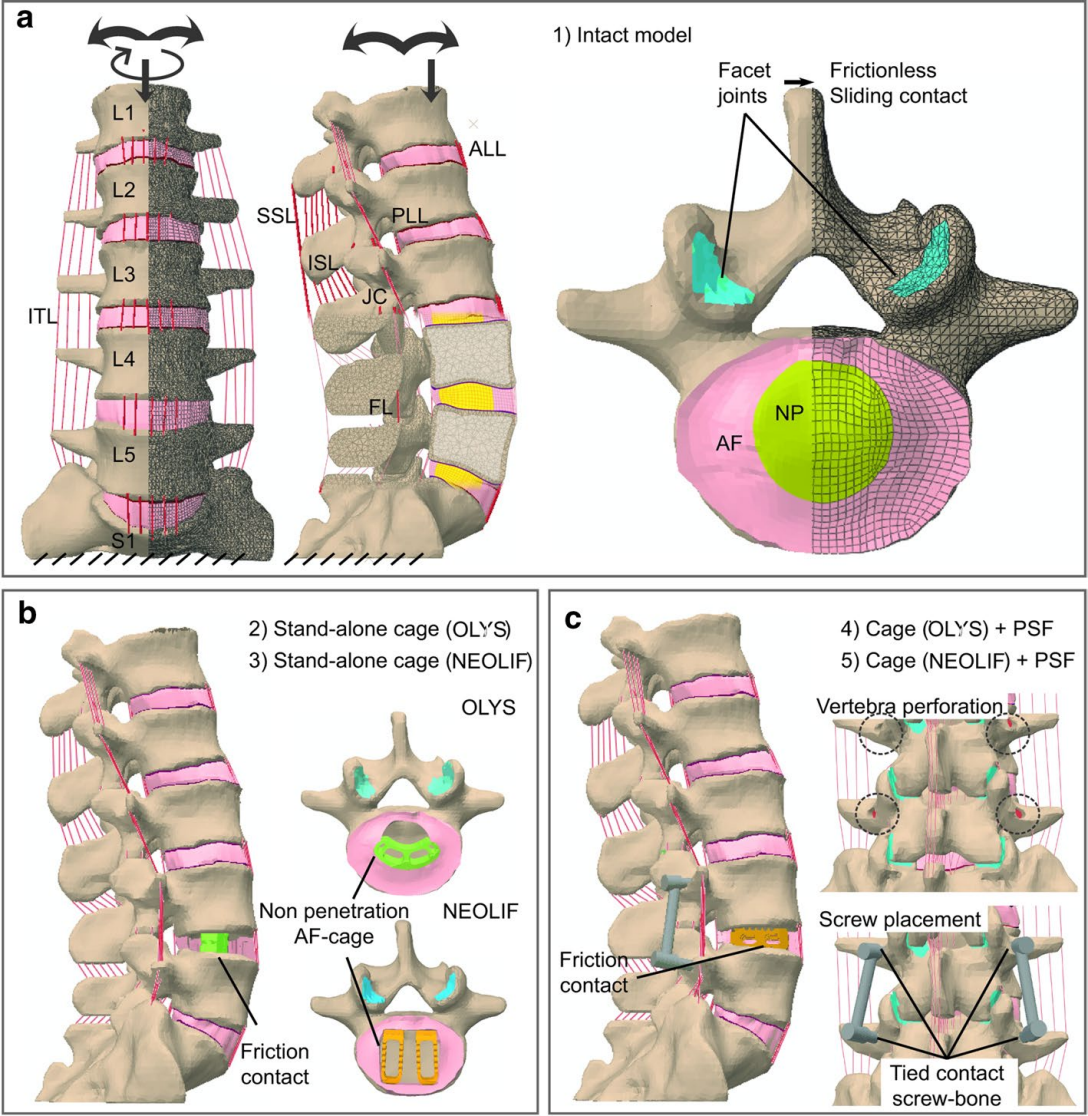
FE model of the lumbar spine. **a** (1) Intact model (L1–S1). Frontal and lateral view of the whole lumbar spine with a schematic representation of the boundary and loading conditions and the ligaments [antero-longitudinal ligament (ALL); postero-longitudinal ligament (PLL); intertransverse ligament (ITL); interspinous ligament (ISL); capsular ligaments (JC); flaval ligament (FL); supraspinous ligament (SSL)] (left). Top view of L5 and the disc between L4–L5 (right). **b** Stand-alone models. Two different cages were introduced in the L4–L5 interbody space: (2) OLYS cage and (3) NEOLIF cage. A lateral view of the whole lumbar spine and the top view of cages placement are shown. **c** Cage + PSF models. The stand-alone models have been supplemented with PSF after the perforation of L4 and L5 vertebrae: (4) OLYS + PSF and (5) NEOLIF + PSF

A porohyperelastic FE model of the whole lumbar spine (L1–S1) from previous works was used as the intact model [35]. This model was modified to construct four different FE models adding instrumentation as shown in Fig. 1. These models can be summarised as follows: (1) Intact; (2) Stand-alone cage (OLYS); (3) Stand-alone cage (NEOLIF); (4) Cage (OLYS) + PSF; and (5) Cage (NEOLIF) + PSF. All of them were subjected to the same boundary and loading conditions. A total displacement and rotation restriction was imposed on the lower face of the sacrum. Eight hours of free swelling in which the internal fixed charge concentration is equilibrated with the external solution by means of the osmotic pressure was simulated [36]. A preload of 100 N was applied at the centroid of vertebra L1 followed by ± 10 Nm moment load in flexion–extension, lateral bending (LB) or axial rotation (AR) [18, 37, 38]. All simulations were performed using ABAQUS 6.13 (SIMULIA, Providence, RI, USA).

### Intact model

Intact model is briefly described; more details can be found elsewhere [35, 39]. A computed tomography (CT) of the lumbar spine from a healthy patient was used to reconstruct the bone geometry (slices obtained by 0.5 mm intervals of 512 × 512 resolution). After segmentation, soft tissues were modelled according to the anatomical characteristics as shown in Fig. 1a. Vertebral bodies were meshed using linear tetrahedral elements of 2 mm size, and linear poroelastic properties were assigned differentiating between cortical and trabecular bones. The IVDs consisted of annulus fibrosus (AF), nucleus pulposus (NP) and endplates (EP). These were meshed with linear hexahedral elements after a sensibility test to determine a mesh size of 1.5 mm. The EPs were characterised as poroelastic materials, the AF was characterised as anisotropic porohyperelastic material with two families of fibres, using the Holzapfel strain energy function, and the NP as anisotropic porohyperelastic neo-Hookean material. The osmotic behaviour was included in both AF and NP. The constitutive model of the IVD, as well as the annulus fibres orientation (± 30°), was implemented in an UMAT user subroutine [35]. The seven spinal ligaments (see Fig. 1a) were modelled as uniaxial truss elements with strain-dependent behaviour under traction and without resistance to compression. Initial ligament pre-strain was set as the initial condition according to the experimental values from literature [32]: 5.3% for anterior longitudinal ligament (ALL) and 4.3% for interspinous ligament (ISL). The facet joints were modelled as frictionless surface-to-surface contact combined with a penalty algorithm for normal contact, with a normal contact stiffness of 200 N/m (see Fig. 1a, right). A 0.2 mm thickness was assigned to the cartilage of the facet joints, which was assumed to be linearly elastic and isotropic [40]. All material properties are summed up in Table 1.

**Table 1:** Elastic [*E* and *ν* for elastic material; *C*_10_, *C*_20_, *D*, *K*_1_ and *K*_2_ for a hyperelastic model (Holzapfel strain energy function); *E*_1_, *E*_2_ and *ε*_12_ for bilinear elastic model] and biphasic material properties (*k*_0_: initial permeability, *e*: void ratio, *c*_F,0_: initial fixed charged density, *n*_F,0_: initial porosity) assigned to the different tissues to simulate the spine behaviour in healthy and degenerated (degenerated annulus—Grade IV) state

	Elastic parameters		Biphasic parameters						
	*E* (MPa)	*ν*	*k*_0_ (m^4^/Ns)	*e*	*c*_F,0_ (meq/mm^3^)	*n*_F,0_			
Cortical bone [41, 42]	17,000	0.3	5.77 × 10^−18^	0.05	–	0.05			
Cancellous bone [41, 42]	100	0.2	5.55 × 10^−11^	0.41	–	0.29			
Endplate [41, 42]	20	0.4	7.22 × 10^−13^	4	–	0.8			
Cartilage [40]	35	0.4							
Granular tissue [43]	0.2	0.167							

	Elastic parameters					Biphasic parameters			
	*C*_10_ (MPa)	*C*_20_ (MPa)	*D* (MPa^−1^)	*K*_1_ (MPa)	*K*_2_	*k*_0_ (m^4^/Ns)	*e*	*c*_F,0_ (meq/mm^3^)	*n*_F,0_
Annulus [39, 44, 45]	0.1	2.5	0.306	1.8	11	1.85 × 10^−15^	2.7	1.8 × 10^−4^	0.72
Degenerated annulus [46, 47]	0.45	2.5	0.306	1.8	11	1.45 × 10^−15^	2.4	0.9 × 10^−4^	0.7
Nucleus [39, 44, 45]	0.16	0	0.024	–	–	1.92 × 10^−16^	4.8	2.4 × 10^−4^	0.8

Ligaments [32, 48, 49]	Elastic parameters			Biphasic parameters					
	*E*_1_ (MPa)	*E*_2_ (MPa)	*ε*_12_	Number of elements	Area (mm^2^)	Pre-stress (MPa)			
ALL	7.8	20.0	0.12	10	32.9	0.804			
PLL	1.0	2.0	0.11	9	5.2	0.019			
LF	1.5	1.9	0.062	6	84.2	0.02			
ITL	10.0	59.0	0.18	16	1.8	0.026			
SSL	3.0	5.0	0.2	4	25.2	0.017			

	Elastic parameters			Biphasic parameters					
	Spine level	Stiffness (N/mm)	*ν*	Number of elements	Area (mm^2^)	Pre-stress (MPa)			
JC	L1–L2	42.5 ± 0.8	0.4	14	43.8	0.237			
	L2–L3	33.9 ± 19.2							
	L3–L4	32.3 ± 3.3							
	L4–L5	30.6 ± 1.5							
	L5-–S1	29.9 ± 22.0							
ISL	L1–L2	10.0 ± 5.2	0.4	11	35.1	0.028			
	L2–L3	9.6 ± 4.8							
	L3–L4	18.1 ± 15.9							
	L4–L5	8.7 ± 6.5							
	L5–S1	16.3 ± 15.0							

The pre-stress (equivalent to 5% of space distraction) applied to L4–L5 segment ligaments, in case of stand-alone surgery, is also reported in the last column. The following notation is used for ligaments: ALL—antero-longitudinal ligament; PLL—postero-longitudinal ligament; LF—ligamentum flavum; ITL—intertransverse ligament; SSL—supraspinous ligament; JC—capsular ligament; ISL—interspinous ligament

### Stand-alone cage surgery

Two different commercial cages were modelled using Rhinoceros 5.0 (Robert McNeil & Associates, USA): the first one, commonly used for TLIF (transforaminal lumbar interbody fusion) approach was a single bean-shaped piece (OLYS^®^, Scient’x, Alphatec Spine Inc., France) [50]; the second, normally used for PLIF (posterior lumbar interbody fusion) approach, consisted of two rectangular parallel pieces (NEOLIF^®^, Biomet, Germany) [51]. The geometry of both was extracted from the commercial catalogues provided by the companies which include the most relevant dimensions. Both of them were made of PEEK (*E* = 3600 MPa, *v* = 0.38) [50] and were meshed with tetrahedral elements with a mean element size of 0.5 mm. As a minimally invasive technique was simulated, only the nucleus pulposus was removed from the disc and the rest of the structures (annulus fibrosus, endplates, facet joints and ligaments) remained intact [51]. The cages were placed as shown in Fig. 1b. After the insertion, the empty region left between the annulus and the cage was filled with tetrahedral elements simulating granulation or inflammatory tissue (its mechanical properties are shown in Table 1) [43]. The penetration of the cage into the granulation tissue was avoided with a normal non-penetrating contact as well as the penetration of the cage through the annulus. Surface-to-surface sliding contact with high friction coefficient (0.8) [52] was set in the cage–endplate interface considering the effect of the serrated faces of the cages. The implant size was determined according to the patient spine geometry: 12 mm height for OLYS cage and 10 mm height for NEOLIF. The remaining AF was characterised as Grade IV degenerated tissue based on the Thompson grading system [53] with the material properties outlined in Table 1. A 5% of intervertebral space distraction was considered [19] and the corresponding ligament pre-stress was introduced in the model as the initial condition (Table I).

**Fig. 2:**
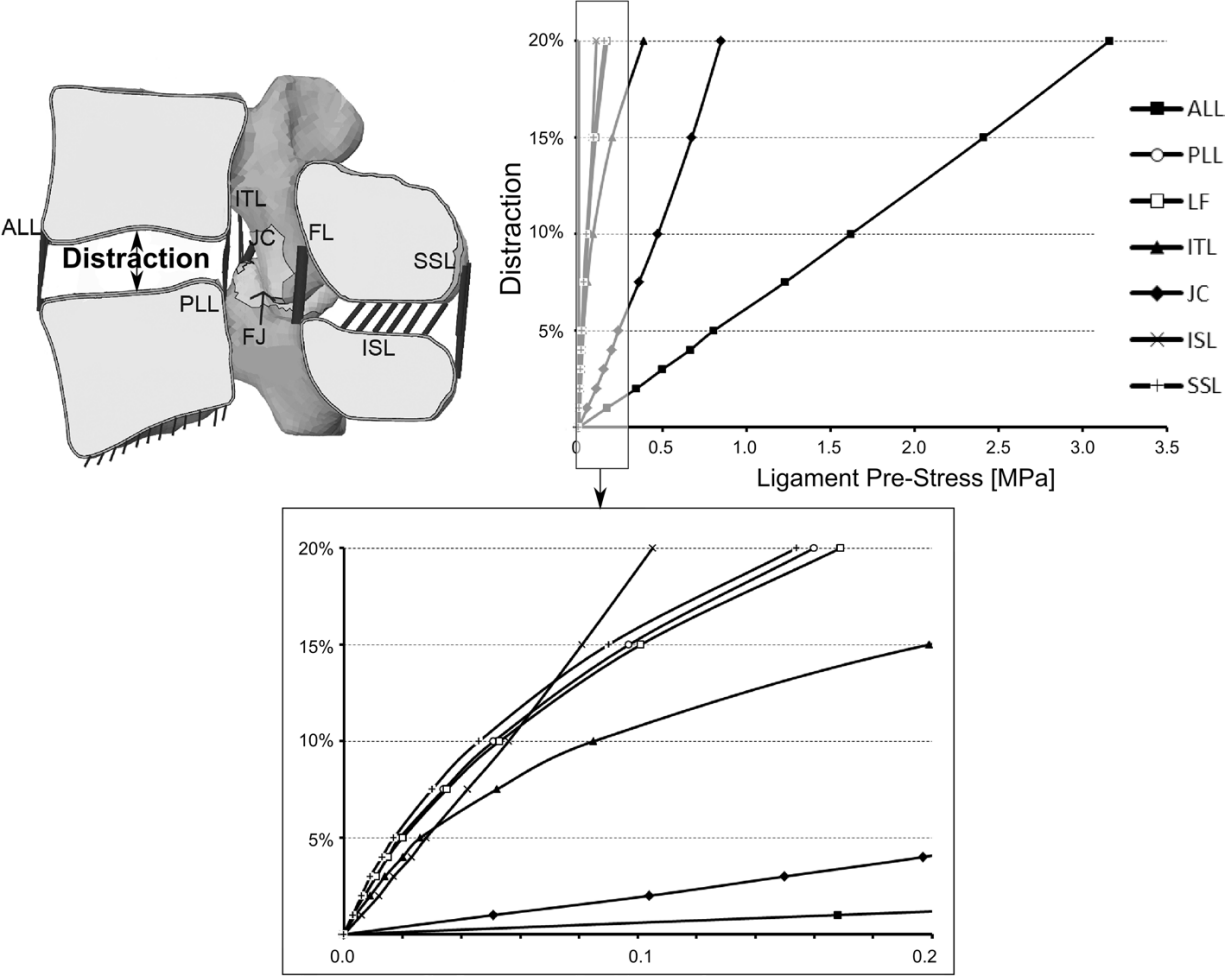
Ligament pre-stress in stand-alone model caused by intervertebral space distraction (from 0 to 20% of the intact IVD height) during cage insertion

In addition, when an intervertebral cage is inserted some intervertebral distraction is exerted to improve the stability of the operated segment [54]. Therefore, here, the sensibility of the lumbar segment motion to ligament pre-stress was studied in an FSU (L4–L5 segment) instrumented with a stand-alone OLYS cage for the same loading conditions. In this case, the bottom surface of L5 was fixed. The FSU motion was tested in all loading directions for distractions ranging from 0 to 20% of the intact disc height, taking into account that each distraction caused a different initial pre-stress of the ligaments. Figure 2 shows the pre-stress value for each ligament depending on the intervertebral space distraction. It can be seen that the highest pre-stress appeared in ALL. Moreover, the non-linear behaviour of the ligaments can be appreciated in the bottom part of the figure.

### Cage insertion with posterior screw fixation

To provide additional stability, the previous models were supplemented with PSF. L4 and L5 vertebrae were perforated before screw insertion as shown in Fig. 1c. A tie contact was assumed at the bone–screw interface. The fixation (diameter of rods and screws of 5 mm) was made of titanium (*E* = 100000 MPa, *v* = 0.33) [55] and meshed with tetrahedral elements of 1 mm size.

## Results

### Validation

The intact model was validated comparing the ROM of each segment with experimental and computational results from the literature [37, 56–58] as shown in Additional file 1. Two different validations were made. First, each segment range of motion was compared to experimental data and computational results found in the literature. This comparison was made for the principal rotations axes (flexion–extension, lateral bending and axial rotation). It was seen that both for flexion–extension movement and lateral bending the correspondence between our results and those of the literature was very high. This was not the case for axial rotation, where more significant differences were obtained.

Furthermore, an additional validation with different moment value was also performed. The more recent study of Campbell et al. [58] was used, and here the total rotation of the lumbar spine (L1–L5 rotation) was evaluated. In this case, the results for extension, lateral bending and axial rotation perfectly fitted within Campbell et al. results. However, the flexion rotation in our simulations was higher.

Therefore, the present finite element model can represent the non-linear behaviour of the human lumbar spine and can serve as a reference to qualitatively analyse the changes when some modification in the mechanical environment of the spine is simulated.

### Movement of the affected segment (L4–L5 segment)

**Fig. 3:**
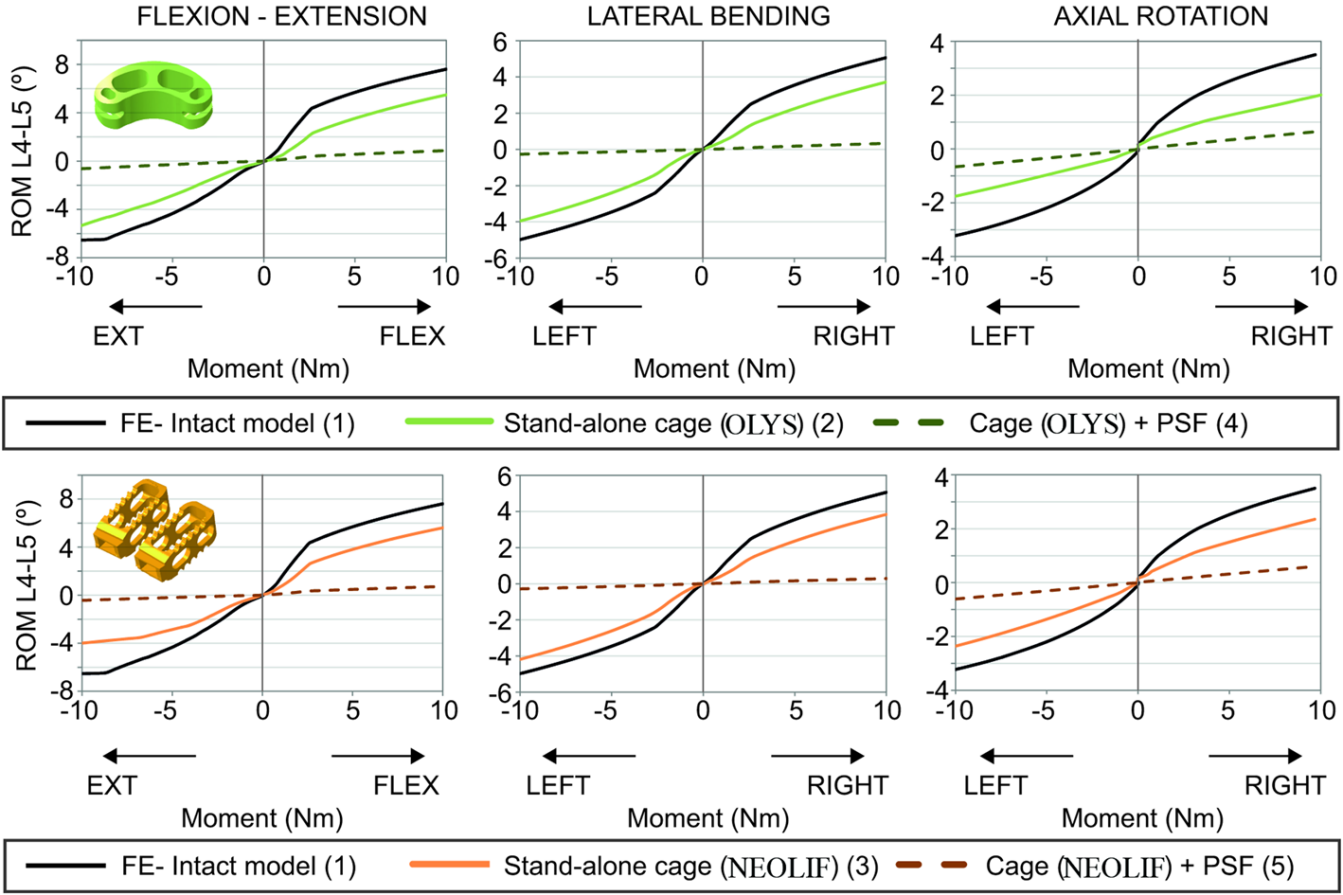
Moment–rotation curves. Range of motion in L4–L5 segments in flexion, extension, lateral bending and axial rotation for OLYS (top) and NEOLIF (bottom) approaches in comparison with the intact movement

Moment–rotation curves were analysed and compared for the five finite element models to see the effect of each surgery over the segment mobility (Fig. 3). When PSF was used, a drastic loss of motion occurred regardless the load direction or the type of intervertebral cage. In these cases, the immobilisation was more pronounced in flexion, extension and LB than in AR. Meanwhile, the stand-alone cages allowed for a wider ROM without exceeding the movement of the intact segment. The stiffness, defined as the moment applied divided by the ROM achieved, of L4–L5 segment in models (2) and (3) was greater in extension and AR movements (around 75%) than in flexion and LB (around 25%). Comparing between cages, the OLYS (2) showed a higher ROM restriction for AR, whereas NEOLIF (3) reduced more the extension rotation. In flexion and LB, the behaviour of the spine with both implants was similar.

**Fig. 4:**
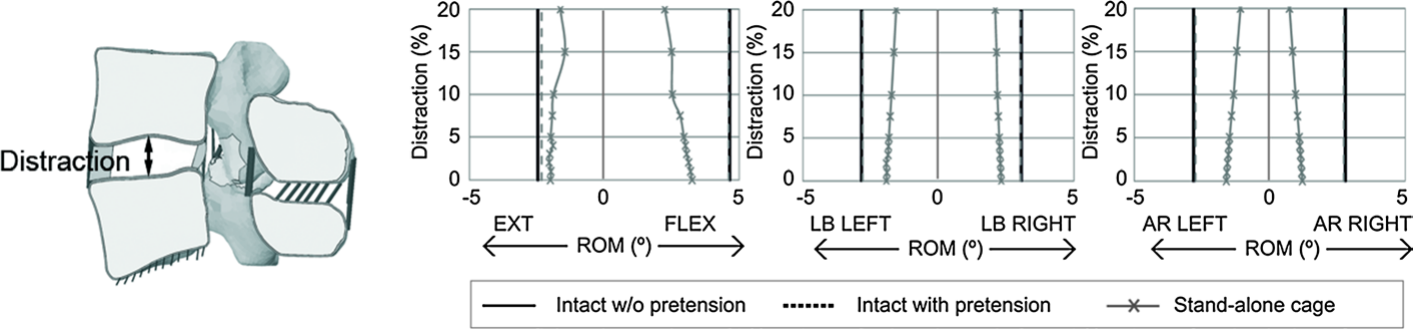
FSU analysis of the influence of ligament pretension. Range of motion of an FSU in which the pretension of the ligaments due to intervertebral space distraction is considered. The value of the distraction is introduced as a percentage of the height of the intact intervertebral disc. The ROM for each moment is shown for an intact FSU with and without pretension, and for an operated segment with a stand-alone cage

On the other hand, as it is common to introduce a cage a little wider than the intervertebral space [54], the influence of the ligament pre-stress was also studied. For that, only an FSU was analysed and different pretension of the ligaments was taken into account. As mentioned previously, Fig. 2 shows the initial stress of each ligament for different space distraction. It was obtained that ALL, JC and, at high distraction levels, ITL were mostly affected by space distraction. The influence of this ligament pretension can be seen in the movement of the FSU (Fig. 4). The segment rotation is shown for an intact FSU with and without considering the initial pretension and for an operated FSU with the initial pretension. The movement was reduced in all loading directions by increasing the distraction. ALL affected the extension movement, whilst JC decreased the rotation primarily in flexion and axial rotation. Lateral bending movements were the less influenced by the pre-stress. For instance, for 10% distraction, flexion and AR ROM decreased around 20%.

### Subsidence risk

The subsidence risk is related to the contact pressures that the cages exerted on the endplates. These pressures appear on the interfaces between cage and bone during loading. When this pressure exceeds the yield stress of the underlying material, a plastic deformation occurs and then the cage can penetrate inside the bone decreasing the intervertebral space height [59].

**Fig. 5:**
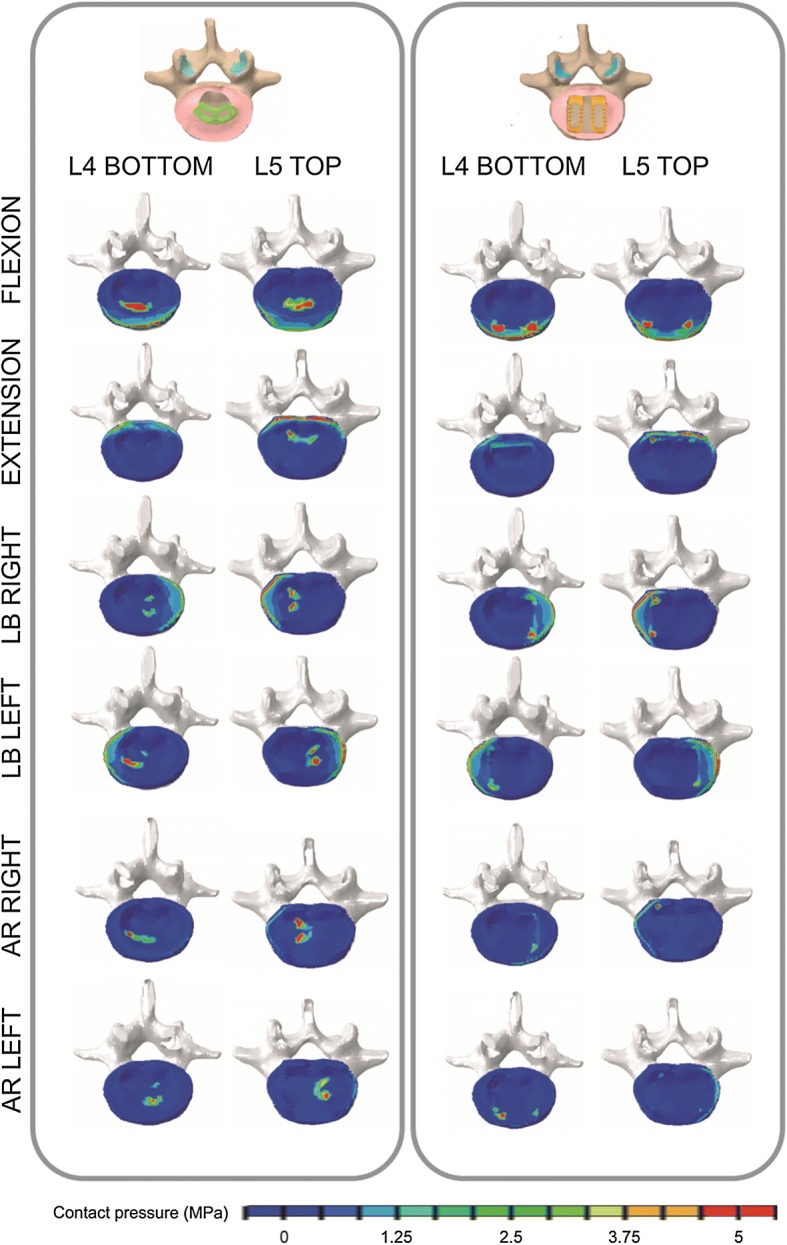
Contact footprints. Contact pressure distribution on the top endplate of L5 and bottom endplate of L4 for both stand-alone cages in each rotation movement

In our calculations, when PSF was introduced, the loads were distributed between the cages and the fixation and therefore the contact pressures were low. The behaviour was totally different for stand-alone cage models. Thus, the subsidence risk was only analysed in finite element models (2) and (3), and the contact pressures for the different movements were evaluated for OLYS and NEOLIF stand-alone cages. These results are shown in Fig. 5. It can be seen that the maximum value of the contact pressure (around 5 MPa) was similar for both the designs.

On the other hand, although there were no significant differences between OLYS and NEOLIF designs, the footprint left by each cage was different. Figure 5 shows the bottom endplate of L4 and the top surface of L5. The contact pressure distribution depends on the analysed movement. For instance for flexion movement, the pressures were distributed on the anterior part of the endplate, whilst for extension these were located on the posterior part. When NEOLIF was used, the pressure was more concentrated at the corners of the cages, whilst with the OLYS cage the contact pressures were distributed in a larger area on the central region. The pressures were in general smaller for the NEOLIF cage for every movement; this result was more obvious for axial rotation.

### Biomechanical changes in operated and adjacent levels

**Fig. 6:**
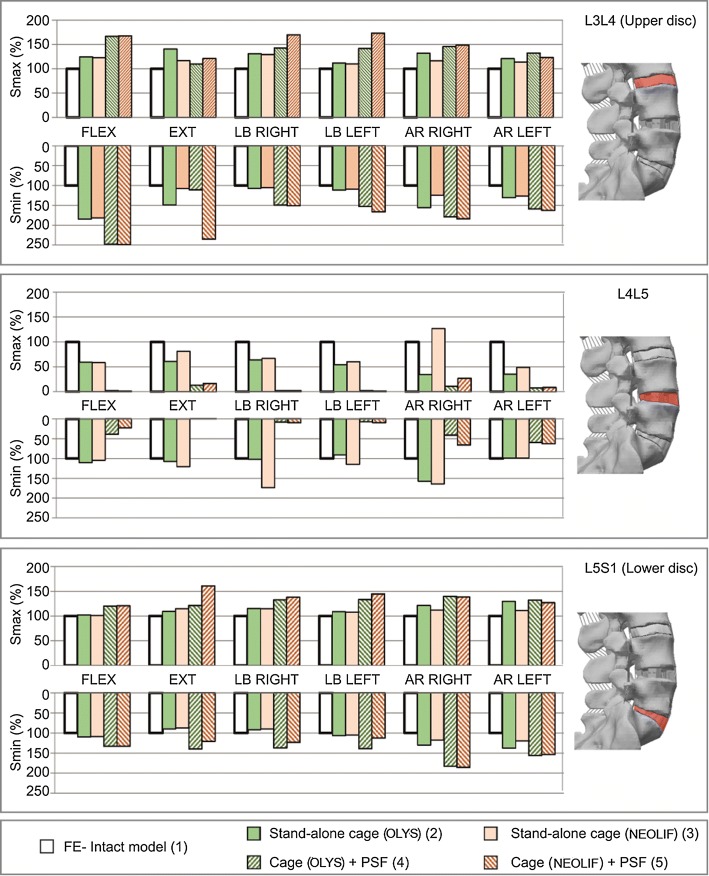
Maximal and minimal principal stresses in the remaining AF (L4–L5) and the adjacent discs (L3–L4 and L5–S1) in flexion, extension, lateral bending (LB) and axial rotation (AR) as percentage of the intact values for each simulated model. For comparison, the stresses were measured at the same IVD location along the models

Finally, in the remaining AF of the operated segment (L4–L5), the maximal and minimal principal stresses almost disappeared when PSF was used in flexion–extension and lateral bending, and they were reduced to a half in axial rotation, as shown in Fig. 6. On the other hand, when no PSF was added, the AF tissue of the operated segment absorbs a high percentage of the compressive loads and therefore the compressive stresses were higher than those of the intact discs whilst tension stresses were lower. This result can be extended to all loading cases with the only exception of NEOLIF cage in right rotation.

Attending to the adjacent segments, PSF caused a higher increase of principal stresses in both the segments as shown in Fig. 6. In flexion, the increase in stresses was more pronounced in the upper adjacent disc than in the lower one. However, in LB and AR, the influence over upper and lower discs was similar but depends on the load direction and the type of cage. When there was no posterior fixation, the stresses in the adjacent discs experienced a minor increase but it was also higher in the cranial disc than in the caudal disc for every movement.

## Discussion

The main goal of this work was to compare between two different intervertebral lumbar cages with and without supplemental PSF and argue if stand-alone cage is a feasible solution for lumbar disc degeneration and hernia.

First, the intact finite element model was validated. In general, a good accordance was obtained between our results and those of the literature. For the case of axial rotation, the differences were higher. However, these disagreements were also obtained by the numerical simulations of Park et al. [57] and they can be related with the tissues involved in the cadaveric specimens of Panjabi et al. [37], which are not considered in the computational models. Moreover, the discrepancies for every movement were higher as the rotation was measured in the most caudal level of the spine. This can be explained by the fact that constraints were applied in S1. Another explanation could be related with the ligaments behaviour. We have to take into account that ligaments are rather different from upper to lower levels with different area and mechanical properties. However, due to the lack of data in the literature, we used the same definition at all levels, with exception of JC and ISL ligaments.

Attending to the operated spines [models (2) to (5)], the results revealed a drastic reduction in ROM when PSF was used whilst stand-alone cages allowed for a greater ROM. Considering that spine instability occurs if the ROM of the affected segment exceeds that of the intact segment for the same moment load [60], in our simulations, all the different models (with and without posterior fixation) showed that all the segments were stable since the stiffness increased in all cases. However, PSF achieved a much more stable union in all cases. The movement of the operated segment, although it remained stable, was high and therefore this type of surgery might compromise the complete fusion of the vertebra. Nevertheless, stand-alone cages have proven to be sufficient for intervertebral fusion when used in combination with bone graft [2, 13, 61]. Furthermore, when no graft is added, a fibrosis occurred around the implant preventing from migration and preserving some segmental motion [51].

Other authors have evaluated the ROM for a variety of cage designs and load magnitudes from the computational and experimental point of view. In vitro studies have reported ROM reductions with stand-alone cages between 6 and 70% of the intact movement in flexion–extension and LB for complete lumbar spines [17, 62] or FSUs [38]. This wide range in experimental findings may be caused by the different cages and surgical approaches used. However, all of them agreed with our results in showing that the restriction in the AR is lower and, in some cases, it was even higher than the intact movement. Moreover, all of them reported a significantly greater segmental stiffness when posterior screw fixation was added. As well as, in vitro studies, FE models from literature also reported a broad range of ROM reduction depending on the cage design and cage material in the whole lumbar spine [22, 63] and FSU [19, 21]. However, as happened in experimental works all of them showed a less stabilisation for AR and stiffer segments with the use of PSF, which is in accordance with the results of this work except in AR with stand-alone cages. In this work, the segment was stiffer in AR, which could be a consequence of ligament pre-stress, especially of the capsular ligaments. Here, the role of ligament pre-stress, due to cage insertion, in the stabilisation of the segment was considered. ALL and JC ligaments, which are dominant under extension [64], were the most affected by space distraction. Consequently, the ROM in extension was reduced with increasing distraction. The capsular ligaments also restricted flexion and AR movements, providing additional stability.

Apart from stability, the interaction between cage and endplate was studied. Cage subsidence is one of the most common causes of failure in lumbar surgeries [13, 29] and contact pressure can be related to this phenomenon [65]. The loss of disc height due to subsidence (i.e. the device sinking through the endplate) causes loss of correction in approximately 30% of the operated cases [59]. For PSF models, the contact pressures were very low and therefore it can be assumed that there would not be risk of subsidence. However, for the stand-alone constructs the behaviour was different, since the stiffest part of the model, which now corresponds to the stand-alone cage, absorbs most part of the loading. The pressure values was similar for both the designs (OLYS and NEOLIF), and this value should be compared with the yield stress of the endplates. Patel et al. [59] found that the maximum tolerable pressure of the endplates has a median value of 6.7 MPa. Our contact pressures were slightly lower than this value; however, the pressures were very near the median value and therefore, it would suggest a risk of subsidence of these implants. The OLYS cage showed a homogeneous contact pressure distributed in a large contact area. On the contrary, the NEOLIF cage exhibited concentrated contact pressures at the cage edges, as shown in other studies [52, 63]. However, whilst OLYS cage laid in the central part of the endplate, NEOLIF contact pressures were located in the outer part of the bony endplate, where its strength is higher [66]. It is known that subsidence risk depends on bone properties and is different for each patient [8, 67, 68]. Here, it was obtained that the contact pressures were near the maximum tolerable pressure of the bony endplates, so a deeper analysis including the local strength of the bony endplate would be necessary to discuss which cage would be more likely to subside.

Finally, the stresses’ distributions in the affected and adjacent segments were analysed. The addition of PSF reduced the maximal and minimal principal stresses in the operated annulus by more than 50%. The stand-alone construct also caused a reduction in the maximal stresses in the AF around 30%; however, the minimal principal stresses were increased in some movements. Additionally, it has been hypothesised that the addition of PSF could lead to the development of IVD degeneration in the segments adjacent to the fused level due to alterations in the stress–strain distribution [69–72]. In this work, a greater increase in tension and compression stresses was reported for the finite element models with PSF, whilst the stand-alone cages slightly altered the stress distribution in the adjacent segments, which has been also seen in the literature [73]. Moreover, the influence of PSF was higher in the cranial segment than in the caudal one, because the changes on stresses were more significant in the upper segment. Changes in the biomechanical environment of biological tissues (by means of changes on the value or/and distribution of the stresses) can cause damage to these tissues [74]. This result is in accordance with Sears el al. [75], who found that the reoperation for adjacent segment disease occurred more frequently at levels cranial rather than caudal to L4–L5 fused segments.

Although special care has been taken in computationally reproducing the physiological behaviour of the tissues and the events after the surgery, this work has several limitations. Despite spinal ligaments exhibit a non-linear, anisotropic and viscoelastic response [32, 48, 76, 77], they have been simulated as non-linear uniaxial elements. A shell or 3D model of the ligaments would allow the implementation of a more realistic material behaviour of these tissues including their preferential collagen fibre orientation. Furthermore, few experimental data are available regarding spinal ligament pretension. For deeper studies, more experimental work is needed. With respect to the exact geometry of the intervertebral cages, some simplifications were made. In the cage of OLYS implant, the top and bottom surfaces of the cage present grooves on three small zones to avoid retropulsion. These grooves were not included and a high friction coefficient was used instead, and therefore, the overall behaviour of the spine would not be affected. Regarding the PSF, a tie contact was defined at bone–screw interface. A high friction coefficient due to the threads of the screws could have been defined. However, a penalty formulation for contact definition between these elements only would affect the stress distribution in the bone around the screw, but not the movement of the screw inside the bone and, at the same time, would increase the computational time. Therefore, the assumption of a tie contact would be a valid simplification to study the intersegment movement and stresses in the intervertebral discs. Finally, only quasi-static loads were applied to the models. For a more accurate evaluation of the surgical technique, cyclic and impact loading should be considered.

To conclude, when should stand-alone cages be considered instead of traditional PLIF surgery (cage + PSF)? Some authors [78] consider that stand-alone cages would be used in young patients with discogenic pain originating from L4–L5 and/or L5–S1 and no major degenerative changes in the posterior column due to the possible risk of adjacent segment disease associated with PSF. Moreover, Costa et al. [1] argue that when placing stand-alone cage, the facet joints are preserved and the destruction of the posterior and facet joint ligaments and of the endplates is minimal, conditions that are crucial to successful bone fusion. On the other hand, long-term follow-ups [79] have obtained that PLIF stand-alone cages were associated with good clinical outcomes but although the fusion rate was excellent, maintenance of disc heights and lordotic alignment were not achieved in the long term. Therefore, there are still pending questions. Zhang et al. [80] in a review article reported that there is no relationship between radiographic fusion and recurrence of symptoms with development of subsidence. They even suggested that subsidence may be the process of bone incorporation between cages and endplates. Moreover, these authors also relate the posterior screw fixation with an increased rate of adjacent segment degeneration. The following question could be then formulated: where is the equilibrium point between intervertebral fusion and stabilisation?

In this work, a minimally invasive posterior insertion of an intervertebral cage (OLYS and NEOLIF) was compared using a stand-alone design or adding supplementary fixation. The outcomes of these two techniques were compared, and although stand-alone cage may diminish the risk of disease progression along the spine, the spinal movement in this case might compromise the vertebral fusion.

## Additional file

**Additional file 1.** Moment–rotation curves validation. A) Moment–rotation curves in flexion, extension, lateral bending and axial rotation: comparison of the relative range of motion (ROM) amongst each segment of the intact FE model, in vitro and computational models from the literature [37, 56, 57]. The results of the current model are in agreement with those from the literature but in extension and axial rotation at the lower levels, where the movement was higher. The rotation in these directions was influenced by the contact in the facet joints which is geometry-dependent and may be the cause of the disagreement in the low segments. B) Total rotation of the lumbar spine in comparison with the results of 18 patient-specific models [58]. The total motion is in agreement for extension, lateral bending and axial rotation. In flexion, the total rotation recorded in our FE model was above the range reported by Campbell et al.; however, the segmental ROM matched closely the rest of the studies.

## Data Availability

Not applicable.
