# Supplementary figures and images for: Is there any advantage of using stand-alone cages? A numerical approach
Source: Biomed Eng Online. 2019 May 22;18:63. doi: 10.1186/s12938-019-0684-8 (PMC6530002; doi:10.1186/s12938-019-0684-8)

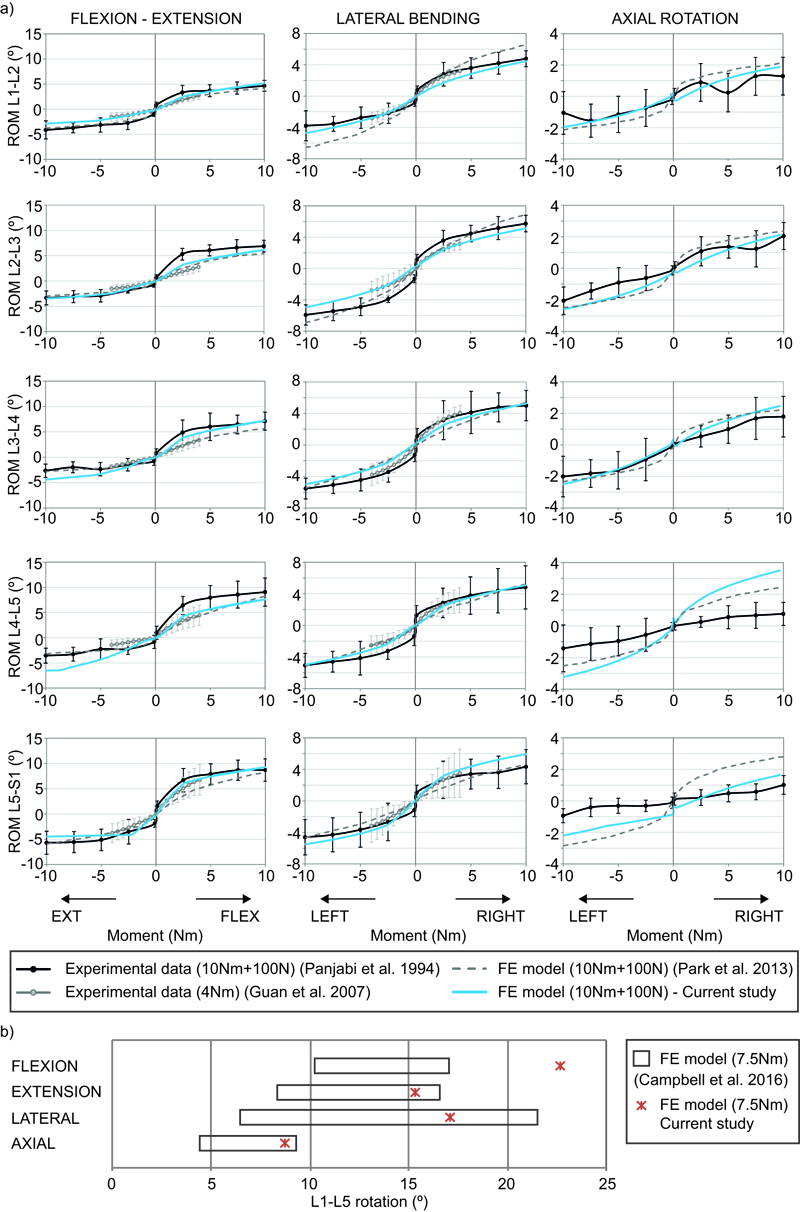

Supplement: Supplementary file 1 — Additional file 1. Moment–rotation curves validation. A) Moment–rotation curves in flexion, extension, lateral bending and axial rotation: comparison of the relative range of motion (ROM) amongst each segment of the intact FE model, in vitro and computational models from the literature [37, 56, 57]. The results of the current model are in agreement with those from the literature but in extension and axial rotation at the lower levels, where the movement was higher. The rotation in these directions was influenced by the contact in the facet joints which is geometry-dependent and may be the cause of the disagreement in the low segments. B) Total rotation of the lumbar spine in comparison with the results of 18 patient-specific models [58]. The total motion is in agreement for extension, lateral bending and axial rotation. In flexion, the total rotation recorded in our FE model was above the range reported by Campbell et al.; however, the segmental ROM matched closely the rest of the studies. [file 12938_2019_684_MOESM1_ESM.tif]
